# Effect of Neuroinflammation on Synaptic Organization and Function in the Developing Brain: Implications for Neurodevelopmental and Neurodegenerative Disorders

**DOI:** 10.3389/fncel.2017.00190

**Published:** 2017-07-11

**Authors:** Amin Mottahedin, Maryam Ardalan, Tetyana Chumak, Ilse Riebe, Joakim Ek, Carina Mallard

**Affiliations:** Department of Physiology, Institute of Neuroscience and Physiology, Sahlgrenska Academy, University of Gothenburg Gothenburg, Sweden

**Keywords:** preterm infant, neuroinflammation, synapse formation, neurodevelopmental disorders, neurodegenerative diseases

## Abstract

The brain is a plastic organ where both the intrinsic CNS milieu and extrinsic cues play important roles in shaping and wiring neural connections. The perinatal period constitutes a critical time in central nervous system development with extensive refinement of neural connections, which are highly sensitive to fetal and neonatal compromise, such as inflammatory challenges. Emerging evidence suggests that inflammatory cells in the brain such as microglia and astrocytes are pivotal in regulating synaptic structure and function. In this article, we will review the role of glia cells in synaptic physiology and pathophysiology, including microglia-mediated elimination of synapses. We propose that activation of the immune system dynamically affects synaptic organization and function in the developing brain. We will discuss the role of neuroinflammation in altered synaptic plasticity following perinatal inflammatory challenges and potential implications for neurodevelopmental and neurodegenerative disorders.

## Introduction

**Figure 1:**
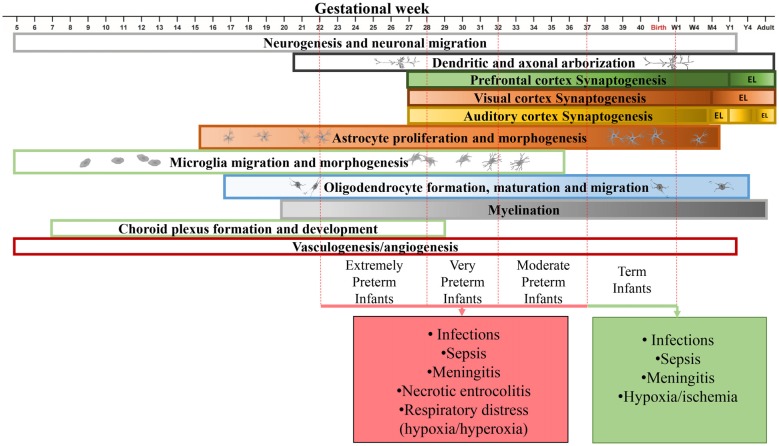
Time line of human brain development in the perinatal period. Developmental time course of neurons (Lenroot and Giedd, 2006; Budday et al., 2015; Paredes et al., 2016), synapses (Huttenlocher and Dabholkar, 1997; Glantz et al., 2007), astrocytes (Roessmann and Gambetti, 1986; Budday et al., 2015), microglia (Esiri et al., 1991; Monier et al., 2007), oligodendrocytes (Rakic and Zecevic, 2003; Yeung et al., 2014), myelination (Tosic et al., 2002; Yeung et al., 2014), choroid plexus (Dziegielewska et al., 2001) and vasculature (Budday et al., 2015) in the human brain. Major complications during the perinatal period are shown. W1, week 1; M4, month 4; Y1, year 1; EL, elimination.

The theory by Hebb postulates that: “Cells that fire together, wire together” (Hebb, 1949). Thus, what determines “who” we are, in part, relies on how our brain is anatomically wired. Brain plasticity involves structural and functional refinement and remodeling of neural connections at the synapse. These processes occur throughout life, but are particularly extensive and pivotal in the prenatal and postnatal period (Merzenich et al., 2014; Figure 1). Brain development begins with an intricate process of neural cell generation, differentiation and migration, which is regulated intrinsically by expression of transcription factors as well as extrinsically by extracellular signals or morphogens (Colón-Ramos, 2009; Lui et al., 2011). This is followed by neuronal axonal growth, which is guided by various attractive and repulsive cues and is regulated by local mRNA translation (O’Donnell et al., 2009; Holt and Schuman, 2013). Synapses are formed when the target synaptic partners are identified by the axons and then finally pruned to sculpt the neural circuits (Colón-Ramos, 2009; Riccomagno and Kolodkin, 2015). The functions of these connections are further refined by synaptic plasticity.

The perinatal period constitutes a critical time in central nervous system (CNS) development, during which pregnancy, delivery and neonatal complications impose a risk of injury to the developing brain. Among these complications are chorioamnionitis, preterm birth, neonatal infection, meningitis, neonatal hypoxia-ischemia, neonatal stroke, neonatal respiratory distress syndrome and necrotizing enterocolitis. Although the etiology is diverse, inflammation is a common factor in many of these conditions (Hagberg et al., 2015). According to World health organization (WHO), the rate of preterm birth ranges from 5% to 18% of live births depending on geographical location^1^. Preterm birth is often associated with fetal infection due to transmission of pathogens from the mother to fetus or hematogenic routes (Goldenberg et al., 2008). *Escherichia coli* and group B streptococci are the leading infectious agents in early neonatal infections, while coagulase-negative staphylococci are responsible for at least 50% of late onset neonatal sepsis (Mallard and Wang, 2012; Strunk et al., 2014). Both culture-positive infections and clinical sepsis (i.e., signs of infection but without positive microbial cultures) in infants born preterm increase the risk for white-matter brain injury (Chau et al., 2012). Preterm infants are at high risk of life-long neurodevelopmental impairment manifesting as cognitive or learning difficulties, behavioral problems, or sensory deficits (Stoll et al., 2004; Mwaniki et al., 2012; Spencer and Meyer, 2017). Meta-analysis of studies of very preterm infants with infection demonstrated that necrotizing enterocolitis and meningitis were most strongly associated with impairment in mental and motor development (van Vliet et al., 2013). The effects of preterm birth and infection on neural cell proliferation and survival, axonal damage and myelination have been widely studied in different experimental models (Fleiss and Gressens, 2012). It is clear that microglia, the professional immune cells in the brain, are important in the pathogenesis of injury to the immature brain (Pierre et al., 2017). However, less is known about how microglia activation following perinatal infection/inflammation influences brain plasticity. Recent studies suggest that microglia are critical in synaptic pruning processes in the healthy developing brain (Paolicelli et al., 2011). Whether perinatal conditions that alter microglia function affect synaptic pruning is an important question, which has only begun to be addressed.

## Neuroinflammation in The Developing Brain

Both antenatal and postnatal challenges are associated with inflammation in the brain, including microglia activation, astrogliosis and increased expression of pro-inflammatory cytokines (Supramaniam et al., 2013; Baburamani et al., 2014). Neuroinflammation is not only a hallmark of infections, but also occurs secondary to non-infectious events such as cerebral hypoxia-ischemia (Hedtjarn et al., 2004). Thus, in the following sections we will discuss both non-infectious and infectious neuroinflammation.

### Non-Infectious Neuroinflammation

In newborn infants, increased levels of cytokines in the cerebrospinal fluid (CSF) correspond to the degree of hypoxic-ischemic encephalopathy (HIE; Savman et al., 1998). HIE is commonly modeled in neonatal mice by combining cerebral ischemia (reduced brain blood flow) and hypoxia (reduced blood oxygen), which result in brain pathologies relevant for the human condition (Rice et al., 1981). Using this model, transcriptomic analysis showed an extensive inflammatory response in the brain that lasted for at least 3 days (Hedtjarn et al., 2004). Also, in large animal models of fetal asphyxia, induced by umbilical cord occlusion in the midgestation fetal sheep, neuroinflammation has been detected as indicated by microglia with an amoeboid phenotype, which corresponds to activated microglia (Mallard et al., 2003). Flow cytometry analysis of brain tissue has shown that there is a large expansion of the total microglia pool after neonatal hypoxia-ischemia in mice, which is characterized by mixed cell populations expressing both classical and alternative activation markers (Hellström Erkenstam et al., 2016). Although it is likely that different types of microglia have different roles after hypoxia-ischemia, certain microglia-produced cytokines, such as IL-18, clearly play an important role in the pathogenesis of brain injury after neonatal hypoxia-ischemia (Hedtjarn et al., 2002). On the other hand, depletion of microglia is deleterious in perinatal ischemic brain injury models (Faustino et al., 2011).

The role of astrocytes in ischemic injury is complex and it is still unclear whether astrocyte reactions are beneficial or detrimental to the brain (Pekny and Pekna, 2014). One of the hallmarks of astrocyte response to injury is upregulation of the glia fibrillary acidic protein (GFAP), which is the main constituent of astrocyte intermediate filaments. Transgenic mice that lack GFAP show attenuated reactive gliosis after neonatal hypoxia-ischemia, but without affecting brain injury volume. However, these animals show an increase in the number of surviving newborn neurons in the dentate gyrus after hypoxia-ischemia, suggesting that neonatal mice with reduced reactive gliosis have a more permissive environment to neurogenesis after injury, at least in the hippocampus (Jarlestedt et al., 2010). On the other hand, recent evidence demonstrates that astrocyte-produced cytokines, such as leukemia inhibitory factor, stimulate the regenerative response of neural stem cells in the subventricular zone of the brain after neonatal hypoxic-ischemic injury (Felling et al., 2016). Therefore, astrocytes appear to have important functions in regulating neuronal proliferation after injury, though the specific effects may be region dependent.

In summary, the glia responses after non-infectious hypoxia-ischemia brain injury are multifaceted and include both beneficial and detrimental effects that are time and context dependent. In order to manipulate glia responses with the aim to improve outcome after hypoxia-ischemia, we will need further studies to clarify the microglia and astrocyte specific roles in repair and injury mechanisms.

### Neuroinflammation after Fetal Infection

Clinically, elevated plasma cytokine levels in the fetus, a condition termed fetal inflammatory response syndrome (FIRS), is a risk factor for both short-term morbidity and long-term neurological sequelae (Goncalves et al., 2002; Yoon et al., 2003). In support, a large body of literature has shown that experimentally induced systemic infections lead to inflammation in the brain. Innate immune receptors, such as toll-like receptors (TLRs), are important sensors and mediators of microbial infections and have also been shown to be critical in inflammatory processes in the immature brain (Mallard et al., 2009). Therefore, TLR agonists are often used experimentally to mimic infectious challenges in animals. Convincing evidence shows that systemic TLR activation in the fetus, either by intravenous injection or via amnion, infers phenotypic changes in glia cells in the brain. Fetal sheep subjected to the TLR4 agonist lipopolysaccharide (LPS) through an intravenous bolus infusion at midgestation (Dean et al., 2011) or via the uterine artery late in gestation (Hutton et al., 2008), have increased microglia numbers in the days after the challenge as well as an increased proportion of cells with a reactive amoeboid phenotype. Also, more remote infectious exposure during pregnancy induces a fetal brain inflammatory response. In fetal sheep, a single injection of LPS into the amniotic fluid at different ages between 106 or 123 days gestation (equivalent to a moderate-late preterm human infant with regard to brain development) resulted in microglia phenotype changes as well as an increase in astrocyte numbers up to 2 weeks after exposure (Gavilanes et al., 2009; Kuypers et al., 2013). Moreover, rabbits injected with LPS into amnion at the end of pregnancy showed microglia activation, indicated by enhanced PK11195-binding in the brain, as well as more amoeboid-shaped microglia in periventricular areas and hippocampus in the newborn (Kannan et al., 2007).

Thus it is clear that systemic infections in the fetus give rise to phenotypic changes in glia cells associated with neuroinflammation in a number of animal species. However, we lack full understanding of the underlying mechanisms that transfer systemic inflammation to the brain.

### Fetal Neuroinflammation Following Maternal Infection

Effects of maternal infection on fetal central inflammatory responses are age-dependent as shown by differently expressed cytokines in the fetal brain and brain pathology following poly(I:C) (a TLR3 agonist that mimics viral infections) injected at embryonic day (E) 9 compared to E17 in mice (Meyer et al., 2006). In this study, IL-1β and IL-10 protein expression in the fetal brain was reduced, while IL-6 expression was increased after poly(I:C) given at E9, with opposite effects observed when inflammation was induced at E17. Interestingly the increase in cytokine proteins in the fetal brain tissue was not accompanied by parallel increases in the mRNA expression levels of the corresponding genes, suggesting a non-CNS source. In support, poly(I:C) given to pregnant mice at E11 and E15 resulted in no evidence of microglia activation in the fetus (Smolders et al., 2015). Further, in a stereology-based assessment of microglia numbers there was no effect in the fetus following maternal poly(I:C) stimulation at E12.5 (Garay et al., 2013). On the other hand, in another study where rat dams were challenged with LPS at E15 and E16, an increased proportion of iNOS^+^ and IL-1b^+^ microglia was reported, suggesting an increase in activation state of these cells (Cunningham et al., 2013). Based on these studies, the origin of fetal brain cytokines following maternal infection is unclear. In contrast to LPS, the source of fetal brain cytokines after poly(I:C) is likely not to be central, but could potentially have a maternal origin. These data suggest that production of fetal brain cytokines may differ depending on type of inflammatory stimulus and/or age at the time of immune challenge.

### Neuroinflammation Following Neonatal Infection

The newborn infant is highly susceptible to potentially life-threatening infections. In particular, the preterm infant is at risk for early-onset sepsis (i.e., infections in the blood stream) as well as sepsis secondary to prolonged hospital care and invasive treatments (Strunk et al., 2014). Neonatal sepsis is associated with an increased risk of bacterial meningitis, i.e., infection of the protective membranes surrounding the brain (arachnoid membrane, subarachnoid space). Most commonly, meningitis results from hematogenous dissemination of bacteria through the blood–brain barrier (BBB) or blood-CSF barrier into the brain (Koedel et al., 2002; Kim, 2003). Neuroinflammation is evident by increased CSF concentrations of TNF-α, IL-1β and IL-6 in infants and children with bacterial meningitis (Krebs et al., 2005). Similarly, in animal studies of neonatal meningitis TNF-α, IL-1β and IL-6 are upregulated in cerebral cortex and hippocampus after injection of *Streptococcus pneumonie* or *Streptococcus agalactiae* into cisterna magna in neonatal rat pups (Barichello et al., 2011). The peak of cytokine production coincided with the time of BBB breakdown at 12–24 h after infection (Barichello et al., 2012).

Activation of microglia in neonatal meningitis has been documented in several studies. Flow cytometry analysis of brain tissue showed a 3-fold increase in microglia cell numbers in brain tissue from 6 h to 72 h after intranasal injection of *E. coli* in postnatal day (P) 3 mice (Mittal et al., 2011). In an animal model of meningitis at term, intracisternal injection of *S. pneumoniae* in P11 rats led to microglia activation detected as increased expression of isolectin-4 and galectin-3 in the hippocampus along the disease progression up to 72 h after injection (Bellac et al., 2007). Further, in a pediatric rabbit model, MRI analysis revealed intense microglia activation in the white matter 21 days after subarachnoid injection of *Mycobacterium tuberculosis* at P4–8 (Tucker et al., 2016). Early life *E. coli* infections in rats have also been shown to prime the brain to a second immune challenge with LPS in adulthood (Bilbo et al., 2005). This combined immune exposure caused memory impairment, which was associated with persistent microglia activation as indicated by higher CD11b^+^ and IL-1β expression in the adult brain (Williamson et al., 2011). In experimental studies using TLR agonists as infectious agent, we have shown that systemic injection of LPS to neonatal P5 mice leads to a transient proliferation of CNS resident Iba1^+^ microglia. These cellular changes were associated with persistent phenotypic alterations in microglia characterized by a significant increase in mRNA expression of iNOS and decreased expression of IL-6 2 weeks after the initial LPS exposure (Smith et al., 2014). Also, prolonged administration (P3–P11) of a TLR2 agonist (PAM_3_CSK_4_) to mice acutely elevated levels of IL-1β, IL-6, the chemokine (C-X-C motif) ligand 1 (CXCL1) and monocyte chemoattractant protein-1 (CCL2) in the brain and resulted in adverse brain development as well as changes in microglia density at P50 (Du et al., 2011).

Although somewhat less studied, astrocytes also produce cytokines after neonatal systemic immune stimulation. Following poly(I:C) challenges in P2–P6 mice, astroglia proliferate and upregulation of cytokine genes were observed together with altered spine/dendrite complexity and memory impairment later in life (Ibi et al., 2013). LPS given to 9-day old mouse pups on the other hand reduced survival of dividing astrocytes (Jarlestedt et al., 2013).

Thus, while data on astrocytes currently are inconclusive, microglia-dependent inflammation has convincingly been demonstrated in numerous studies following immune challenges during the neonatal period. In addition, we recently discovered that systemic activation of TLR2 in neonatal mice resulted in a marked increase in neutrophils and monocytes in CSF (Mottahedin et al., 2017a), which was associated with increased vulnerability to hypoxia-ischemia (Mottahedin et al., 2017b). Thus, contribution of peripheral immune cells must be considered when studying the inflammatory responses in the neonatal brain. An important advancement in the field will be to better understand under which conditions circulating cells enter the brain and to what extent peripheral vs. central immune cells contribute to injury.

## Synaptic Organization during Brain Development

The immune system is important for modeling neural circuits, both under normal physiological conditions and in disease (Yirmiya and Goshen, 2011). The neural synapse transmits information between neurons and is critical in shaping neural circuits. Brain synapses constitute a presynaptic axon bouton, a synaptic cleft and a post-synaptic compartment (Garner et al., 2000). Synapses are formed when axonal boutons come in close proximity to a potential post-synaptic partner (Colón-Ramos, 2009). The location and specificity of synapses are determined by guide cues within and outside the axons and dendrites. Among intrinsic cues are surface proteins such as cadherins, neurexins and immunoglobulins (de Wit and Ghosh, 2016). Extrinsic guides include neural derived secreted factors such as netrin and Wnt, and glia derived molecules such as thrombospondins (Christopherson et al., 2005).

Most of what we know about the time course of human brain synaptogenesis is based on the pioneering work by Peter Huttenlocher (Walsh, 2013). Using electron microscopy and thorough synapse counting he discovered that synaptogenesis in the human starts in the fetus. Postnatally, the number of synapses first continue to grow and later begin to decline until reaching stability in infancy or adulthood, depending on the brain region (Huttenlocher and de Courten, 1987; Huttenlocher and Dabholkar, 1997). It was demonstrated that in the human substantia nigra, synapses start to form by week 12 of gestation and become mature by week 15–16 (Sailaja and Gopinath, 1996). The synapse density reaches its peak in human auditory cortex by 3 months of age while in prefrontal cortex this occurs by 3.5 years of age. The number of synapses decline in auditory cortex and becomes stable by the age of 12, while elimination of synapses in prefrontal cortex continues to mid-adolescence. The synaptogenesis timing in visual cortex is similar to that of the auditory cortex, except for that the auditory cortex demonstrates a bi-phasic postnatal synaptic elimination process (Figure 1; Huttenlocher et al., 1982; Huttenlocher and de Courten, 1987; Huttenlocher and Dabholkar, 1997).

In the mouse neuropil, the number of synapses increases postnatally, reaching its peak on day 32 after birth and declines afterwards. However, the constant increase in the total number of synapses during the postnatal period does not preclude the possibility of concurrent elimination of unnecessary synapses (De Felipe et al., 1997). In fact, dendritic spines in the mouse brain cortex are pruned constantly although the proportion of transient spines is larger in younger mice (Holtmaat et al., 2005). Altogether, these human and mouse data suggest that a significant proportion of synapses are pruned postnatally and during early infancy, making it a critical period for development of neural circuits.

## Role of Glia Cells in Synapse Pruning during Development

Synapse pruning should be distinguished from axon pruning. Although the two processes have some common regulating pathways, the former usually precedes the latter (Riccomagno and Kolodkin, 2015). The common pathways include caspase-mediated self-destruction, the proteasome-ubiquitin pathway, repulsive guidance cue signaling (e.g., semaphorins) and neural activity (reviewed in Riccomagno and Kolodkin, 2015). Here, we discuss synapse pruning mechanisms with focus on the role of glia cells, a field where knowledge has advanced markedly in recent years.

In the human fetus, microglia accumulate in brain regions where the first synapses are detected (Verney et al., 2010). One of the first clues of a potential involvement of glia cells in synapse elimination emerged from the electron microscopy observation that microglia and astrocytic processes are in close proximity to the synapse being eliminated after injuries to axons and hypothalamic neurons (Chen, 1978; Tweedle and Hatton, 1984). However, it took a few decades before the interaction was confirmed experimentally in animal models and mechanisms began to be revealed. First, it was shown that immature astrocytes mediate plasticity in visual cortex, as transplantation of neonatal astrocytes into adult cat cortex induced visual dominance plasticity after light deprivation (Müller and Schwarz, 2006). Later, it was shown *in vitro* that neurons co-cultured with glia cells develop more efficient synapses and stronger action potential than neurons alone suggesting a trophic role for glia cells (Pfrieger and Barres, 1997). Likewise, addition of astrocytes to retinal ganglion cell (RGC) cultures significantly enhanced pre-synaptic and post-synaptic function, increased the total number of released synaptic vesicles in response to increased extracellular K^+^, and increased the number of synaptic puncta (Ullian et al., 2001). However, the mechanisms of developmental synapse pruning and involvement of glia cells remained elusive until a ground breaking study published by Stevens et al. (2007) revealed a role for the complement system and astrocytes in synapse elimination. They studied the visual neural pathway as an *in vivo* model of synaptic plasticity and found that the dorsolateral geniculate nucleus (dLGN), that receives input from RGCs, undergoes substantial synaptic remodeling in order to segregate the right and left eye during development (Hong and Chen, 2011). It was found that the complement system components C1q and C3 were upregulated in RGCs of neonatal mice, but not adult, which was mediated by astrocytes. Strikingly, anterograde tracing of RGCs showed a diffuse pattern of projections into the dLGN with defective synapse elimination and eye-specific segregation in adult C1q and C3 knockout mice (Stevens et al., 2007). The same research group later discovered that microglia cells phagocytose and eliminate the RGC inputs in postnatal mouse brain, a process which was dependent on neural activity and complement C3 (Schafer et al., 2012). In further experiments, it was demonstrated that not only microglia but also astrocytes are important in synapse elimination in the developing dLGN. Astrocytes mediate synapse elimination in a transforming growth factor (TGF)-β dependent manner (Bialas and Stevens, 2013) and are also directly involved in synapse engulfment and pruning by using two phagocytic receptors (MERTK and MEGF10; Chung et al., 2013). MERTK has previously been shown to regulate rearrangement of actin cytoskeleton after phagocytosis (Wu et al., 2005), while MEGF10 was shown to mediate axon pruning by glia cells in flies and phagocytosis of apoptotic cells in worms (Wu et al., 2009). Further, the TGF-β1/CaMKII pathway has been identified as a mechanism that contributes to astrocyte control of inhibitory synapse formation (Packard et al., 2003; Diniz et al., 2014).

During this phase of activity dependent pruning, glutamatergic synapses have functional features that are restricted to the developmental period. For example, many synapses have labile AMPA-receptor signaling, such that the synapse quickly loses its functional AMPA receptors upon activation (Xiao et al., 2004; Hanse et al., 2013). This results in a population of synapses being AMPA-silent but with functional NMDA receptor signaling (Isaac et al., 1995; Liao et al., 1995; Durand et al., 1996), a phenomenon that has been described at different developmental stages in many regions of the brain (reviewed in Hanse et al., 2013). The induction of long-term potentiation (LTP) by correlated pre- and postsynaptic activity at these AMPA-labile or AMPA-silent synapses reinstitutes a stable AMPA component in the synapse (Abrahamsson et al., 2008). An AMPA-silent synapse that is not experiencing this form of functional activation has been suggested to be tagged for elimination by the above described pruning mechanisms (for review see Hanse et al., 2013).

Interestingly, a single injection of LPS to P9 neonatal mice (corresponding to the near-term infant in terms of brain development) increased the mRNA expression of C1q, MEGF10 and MERTK, the critical molecules in synapse pruning, in the brain (data extracted from our microarray database; accession number GSE36215; Bolouri et al., 2014). Therefore, it might be plausible that in some neuroinflammatory conditions synapse pruning is dysregulated, leading to excessive pruning. It is also tempting to speculate that the neuroinflammatory response could affect trophic functions of glia cells, leading to alterations of synapse activity and hence the synaptic pruning. This could have long term effects on the function of the neuronal circuitry.

## Effects of Perinatal Challenges on Synaptic Organization

Human data on the impact of perinatal insults on synaptic structure is scarce. Delayed synaptogenesis has been observed in some infants born preterm (Sarnat and Flores-Sarnat, 2016). In the non-human primate monkey, solely preterm birth did not affect the synaptic density in the neuropil, but significantly altered the synaptic size and types (Bourgeois et al., 1989). However, in baboons, preterm birth significantly diminished expression of vesicular GABA transporter, a synaptic marker for inhibitory neurons, in the auditory brainstem (Kim et al., 2014). In the midgestion fetal sheep, cerebral hypoxia-ischemia caused impaired dendritic arborization and synapse formation (Dean et al., 2013; McClendon et al., 2014) and intra-amniotic inflammation in fetal sheep, induced by LPS, activated microglia and astrocytes and decreased pre-synaptic vesicle density (Kuypers et al., 2013). Moreover, systemic inflammation in neonatal rats altered synaptic morphology and significantly downregulated the pre-synaptic vesicle protein synaptophysin (Han et al., 2017). Altogether, these data support the hypothesis that perinatal challenges can change synaptic structure and function. However, full comprehension of the underlying mechanisms that link perinatal inflammation and synapse dysfunction is lacking.

## Synaptic Disorganization in Neurological Disorders

Over the past decade, it has been shown that abnormalities in synapse structure as well as functional plasticity of synapses are involved in the pathophysiology of various psychiatric and neurological disorders, both during development and in the aging brain (Garay and McAllister, 2010; Penzes et al., 2011; Pilato et al., 2012; Di Filippo et al., 2015; Muñoz et al., 2016; Ardalan et al., 2017; Han et al., 2017). Further, it has been suggested that maternal inflammation is an important risk factor for schizophrenia or autism in the progeny (Knuesel et al., 2014). Synaptic disorganization is an integral part of these conditions (Ebrahimi-Fakhari and Sahin, 2015) and glia cells are believed to play a crucial role in synaptic organization and hence neuronal circuitry and connectivity. Accordingly, it has been suggested that an interaction between synaptic disorganization and immune function is associated with cognitive vulnerability (Delpech et al., 2015). In the next section, we will review data that link neuroinflammation and altered synaptic plasticity in neurodevelopmental psychiatric and neurodegenerative disorders.

### Neurodevelopmental Psychiatric Disorders

#### Autism Spectrum Disorder

Neurodevelopmental disorders like autism spectrum disorder (ASD) and learning disorders (also known as intellectual disability), comprise a very heterogeneous group of conditions and in the following section we will focus the discussion on ASD (Association, 2013). Patients with ASD show altered social interaction and communication skills and inflexibility in behavior and interests. A common feature in ASD is that symptom onset occurs in early childhood, thus coinciding with brain developmental processes such as the period of synapse elimination and maturation. ASD have a high component of heritability (about 80%), and intriguingly in familial ASD, mutations in genes associated with synaptic function are common. For example, genetic analysis showed that variants in the genes for postsynaptic cell adhesion proteins neuroligin 3 and 4, and their presynaptic ligand neurexin 1, are linked to ASD (Sudhof, 2008).

Dysregulation of synaptic pruning and changes in neuronal density is thought to result in brain overgrowth, one of the main neuropathological features in ASD (Courchesne et al., 2011). However, the number of studies examining post-mortem brains from patients with non-syndromic ASD at the synaptic level is very small and mostly contains few subjects (for review see Martínez-Cerdeño et al., 2016). One study comparing tissue from 10 ASD subjects with matched controls, (Hutsler and Zhang, 2010) reported an increased number of dendritic spines on Golgi-impregnated pyramidal cells from three different cortical areas. Specifically, spine density was higher in layer 2 of frontal, parietal and temporal cortex and layer 5 of temporal cortex. Similar findings were observed in several model systems where ASD risk genes, like neuroligin-3 and -4, were genetically mutated and inserted into the mouse genome (Penzes et al., 2011; Isshiki et al., 2014). One *in vivo* 2-photon study of spine turnover rates in barrel cortex of three different mouse models of ASD showed a selective increase of spines receiving intracortical input (Isshiki et al., 2014).

Examination of tissue from syndromic ASD also shows changes in spine density and morphology, however with a more complex picture. For example, cortical neurons from patients with mutations in Fragile-X mental retardation 1 (*FMR1*) gene, the best known single gene mutation associated with ASD, have a higher density of spines with more immature morphologic characteristics (Hinton et al., 1991) a finding that has been confirmed in mouse models of the disease (Comery et al., 1997; Irwin et al., 2002; Galvez et al., 2003). Children with Rett syndrome, a rare genetic neurological disorder, often display autistic symptoms. In contrast to Fragile-X patients, cells in frontal cortex from Rett syndrome patients have lower spine density (Belichenko et al., 1994). A majority of individuals that suffer from Rett syndrome carry mutations in a gene encoding for methyl CpG-binding protein 2 (MeCP2). Transgenic mouse models confirm that MeCP2 gene mutations are associated with reduced dendritic complexity and decreased dendritic spine density (Belichenko et al., 2009; Tropea et al., 2009; Landi et al., 2011). Interestingly, a recent prospective neuroimaging study shows that expansion of the cortical surface area occurs as early as 6 months of age and precedes the general brain volume overgrowth in individuals that later develop ASD (Hazlett et al., 2017). Similarly, it has been suggested that early neurodevelopmental events such as excessive proliferation of neural progenitor cells and cell migration herald later effects on dendrites such as decreased pruning (Packer, 2016). Thus, spine density may not be a primary pathology in ASD.

In addition to heritability, environmental factors including maternal infection has been linked to the development of ASD. For example, in a large Finnish cohort (*n* = 1.2 million) there was a significant association between the level of maternal early gestational C-reactive protein (CRP, an inflammatory marker in blood) and autism in the offspring (Brown et al., 2014). In support, maternal immune activation in mice give rise to offspring that display autism-like features, including social and communicative behavior deficiency as well as high level of repetitive behaviors (Malkova et al., 2012). However, the role of microglia-mediated neuroinflammation in ASD is debated. Microglia phagocytic activity has been suggested to be pivotal in protection against ASD in a mouse model of the disease, as transplantation of phagocytosis-competent Mecp2-expressing microglia into Mecp2-deficient mice arrested the disease progress (Derecki et al., 2012). Though, other researchers have not been able to replicate these findings (Wang et al., 2015) and further experiments have demonstrated that microglia contribute mainly to end stages of the disease, effects that were independent of microglia expression of Mecp2 (Schafer et al., 2016). Interestingly, microglia autophagy has recently been suggested to play a role in synaptic refinement and neurobehavioral regulation in ASD. In a transgenic mice model with targeted deletion of the autophagy gene *Atg7* in myeloid cells, there was an increase in dendritic spines and synaptic markers with subsequent behavioral deficits such as social behavioral defects and repetitive behaviors (Kim et al., 2016).

At the functional level, changes in the balance between excitation and inhibition (E/I balance) in the neuronal network have been suggested to be associated with ASD. Alterations in E/I balance can arise as a consequence of changes at multiple levels of the network, including changes in synaptic connectivity, synaptic plasticity and intrinsic excitability of neurons (reviewed in Tatti et al., 2017). For example, a relative increase in excitation over inhibition has been suggested in the Fragile-X mouse model, where, as indicated above, cortical excitatory cells are hyper-connected and there is a reduction in excitatory drive on fast-spiking inhibitory neurons in the barrel cortex (Gibson et al., 2008). The change in neuronal activity might be secondary to alteration in trophic support from glia cells, which can be affected by neuroinflammation. Moreover, this change in neural activity may underlie the dysregulation in synaptic pruning, altogether resulting in a synaptic disorganization and dysfunction observed in ASD.

In summary, dendritic spine density and morphology are common features of ASD and both genetic and environmental factors, such as inflammation, are important causal links to the observed synaptopathy. Although microglia are key players in sculpting the synapse during development, the exact role and timing of neuroinflammation in these processes is debated and need further study. In addition, changes in spine density may be secondary to earlier aberrant neurodevelopmental processes that also should be considered in future studies.

#### Schizophrenia

Schizophrenia is a heterogeneous disorder with a prevalence of 0.5–1% (McGrath et al., 2008). Results of post-mortem studies indicated a decline in the spine density of gray matter particularly in layer 3 of dorsolateral prefrontal cortex (DLPFC), in correlation with lower level of cortical activity (Selemon and Goldman-Rakic, 1999). Studies also provide evidence of smaller size of hippocampus and lower spine density of CA3 dendrites in schizophrenia (Kolomeets et al., 2005). It is unclear what causes the reduction in spine density. However, hereditary factors are likely to play an important role as polymorphisms of the Disrupted in schizophrenia 1 (DISC1) gene is an important genetic abnormality in schizophrenia, resulting in disruption of scaffolding proteins in spines and substantial changes of structural synaptic plasticity (Lipska et al., 2006). Interestingly, transient neonatal disruption of DISC1 signaling resulted in a lack of LTP in adulthood (Greenhill et al., 2015). More importantly, neonatal activation of the immune system by poly(I:C) increased the neurological dysfunction in adult DISC1-impaired mice, suggesting a synergistic effect between neonatal inflammation and genetic risk factors (Ibi et al., 2010). In addition, maternal immune activation, by poly(I:C), was shown to specifically interact with a discrete point mutation of DISC1 and exacerbated schizophrenia-related behaviors in mice, giving further support to the hypothesis that inflammation during development is an important factor in the pathophysiology of schizophrenia and linked to genetic traits (Abazyan et al., 2010; Lipina et al., 2013).

Certainly, among environmental causes, inflammation is a major risk factor in the pathogenesis of schizophrenia (Benros et al., 2011; Anderson et al., 2013; Müller et al., 2015). Blunted type 1 (Th1) and increased type 2 (Th2) immune responses were observed in untreated schizophrenia patients (Müller and Schwarz, 2006). Moreover, increased expression of peripheral benzodiazepine receptors PK11195 and DAA1106, as indicators of microglia activation, has been correlated to schizophrenia positive symptoms and duration of the disease (Van Berckel et al., 2008; Doorduin et al., 2009; Takano et al., 2010). Increased Th2 immune responses can influence synaptic neurotransmission via kynurenine metabolism (Müller et al., 2011). High levels of kynurenine metabolites have been described in CSF and several brain regions in schizophrenics giving support to the hypothesis that synaptic dysfunction following alteration of immune responses is a potential pathway underlying the pathophysiology of schizophrenia (Linderholm et al., 2012).

Both human studies (Westergaard et al., 1999; Buka et al., 2001) and animal models (Ibi et al., 2009; Dickerson et al., 2010) suggest that perinatal exposure to an inflammatory stimulus, such as viral infection, predisposes individuals to schizophrenia. TLR3 is an important sensor of viral infections and it was recently shown that neonatal TLR3 activation results in altered plasticity of dendritic spines several weeks later (at P21), suggesting a long-lasting effect of TLR3 activation on spinogenesis (Chen et al., 2017). Furthermore, maternal activation of TLR3 by poly(I:C) reduced expression of proteins associated with both presynaptic (synaptophysin and bassoon density) and postsynaptic (PSD95 and SynGAP density) function in the hippocampus, some of which persisted into adulthood. These changes were associated with long-term increases in hippocampal IL-1β levels, but without changes in microglia or astrocyte density or systemic inflammation, suggesting a local inflammatory effect in the hippocampus (Giovanoli et al., 2016). Other investigations have also shown that maternal viral infection exposure induces schizophrenia-like alterations of 5-HT_2A_ and mGlu2 receptors in adult offspring (Moreno et al., 2011; Holloway et al., 2013; Wischhof et al., 2015).

Substantial data is available to support an association between perinatal inflammation and development of schizophrenia. Interestingly, some of the genetic mutations identified to affect spine plasticity in schizophrenia (e.g., DISC1) have recently also been linked to perinatal inflammation. Future investigations of these interactive processes will help to better understand the underlying mechanisms of perinatal origins of schizophrenia.

### Neurodegenerative Diseases

While there has been ample studies linking infection during early life with neurodevelopmental psychiatric disorders, as described above, only more recently has evidence emerged to support a role for maternal immune activation in neurodegenerative diseases (Knuesel et al., 2014). In many neurodegenerative disorders, synapse dysfunction is common. Accordingly, in this section, we will discuss similarities between developmental synaptic pruning and pathological synaptic elimination in three neurodegenerative disorders (Alzheimer’s disease (AD), Parkinson’s disease (PD) and Multiple sclerosis (MS)).

#### Alzheimer’s Disease

AD is a progressive disorder characterized by deposition of Aβ plaques and intracellular tau protein neurofibrillary tangles in the brain. Synaptic failure happens in a very early phase during the progression of AD (Selkoe, 2002) and synapse elimination is a neuropathological hallmark in connection with cognitive decline in AD (Scheff and Price, 2006). Suppressed phagocytosis in aging microglia has been suggested to contribute to cognitive decline in AD via overproduction of arginase-1, an enzyme important for l-arginine metabolism (Kan et al., 2015), and anti-inflammatory cytokines, such as IL-10 (Chakrabarty et al., 2015). However, many studies show that AD is characterized by a chronic pro-inflammatory state in the brain, including both astro- and microgliosis, and activation of the classical complement cascade (Akiyama et al., 2000; Glass et al., 2010). *In vitro* studies report increased pro-inflammatory cytokine production by microglia upon stimulation with Aβ-associated plaques (Combs et al., 2001). Evidence also demonstrates that systemic inflammation generated by LPS enhances beta-amyloid generation in the brain, which was associated with cognitive impairment (Lee et al., 2008). Further, microglia may be directly involved in synapse loss as shown in animal models of AD. Using 5xfAD transgenic mice, i.e., animals that accumulate high levels of intraneuronal Aβ42 followed by amyloid deposition, microglia cells were found to mediate pathological synaptic stripping as shown by significant decrease in the number of dendritic spines (Spangenberg et al., 2016). If microglia were removed from these mice, the density of dendritic spines was significantly increased, particularly mushroom and thin spines. Microglia cells have also been shown to be responsible for the early synapse loss in mouse models of AD in a complement-dependent manner (Hong et al., 2016). In a recent study, it was shown that microglia-derived Il-1α, TNF and C1q induced a subtype of reactive astrocytes, so called A1 astrocytes, that is believed to contribute to neuronal death in neurodegenerative disorders, such as AD (Liddelow et al., 2017).

As described before, the complement system is critical for developmental synapse pruning and is markedly upregulated in AD and it has been suggested that the pathological reactivation of developmental C1q-mediated processes drive the disease progression (Stephan et al., 2012). Speculatively, perinatal challenges that induce neuroinflammation might prime the glia cells for such pathological reactivation. Thus, knowledge of how perinatal inflammation affects complement-mediated synapse function may be of great importance for neurodegenerative disorders including AD.

#### Parkinson’s Disease

The main pathophysiology of PD is loss of midbrain dopaminergic neurons, resulting in a reduction of dopaminergic input to the basal ganglia neuronal circuit and cholinergic over activation. The neurotransmitter deficiency contributes to difficulties in learning and memory, and most prominently, to the control of motor function (Calabresi et al., 2006). In advanced PD, the denervation of the dopaminergic nigrostriatal terminals is associated with perturbed synapse plasticity and alteration of striatal dendritic spines (Anglade et al., 1996; Day et al., 2006). In PD mouse models, abnormal spine turnover (i.e., increased elimination and formation) rather than changes in the absolute number of spines has been documented, which was associated with motor cortex dysfunction. These changes in synaptic plasticity were related to abnormal signaling of two main dopamine receptors, D1 and D2 (Guo et al., 2015). Furthermore, changes in glutamatergic signaling have been reported in a primate (Betarbet et al., 2000) and rat model of PD (Picconi et al., 2004).

Both reactive astrocytes and microglia are abundant in the substantia nigra of PD cases indicating a robust inflammatory state (McGeer and McGeer, 2008) and in postmortem tissue from PD patients, microglia express C1q in the substantia nigra (Depboylu et al., 2011). Interestingly, a recent experimental study reports that recovery of striatal dysfunction following theta-burst stimulation is associated with reduced astro- and microgliosis and increased dopamine levels (Cacace et al., 2017). A relatively recent hypothesis is that infections during the perinatal period can predispose for the development of PD in adulthood (Hagberg et al., 2012). In support, animal studies have shown that *in utero* infection increases the vulnerability of adult dopaminergic cells (Ling et al., 2004; Cai et al., 2013). We recently demonstrated that peripheral administration of LPS to neonatal rats induced a sustained increase in IL-1β in substantia nigra up to P70, which was associated with a decrease in the rate-limiting enzyme γ-glutamylcysteine ligase catalytic subunit in glutathione synthesis, and the endogenous anti-oxidant Nrf2 (Patil et al., 2016). Oxidative stress is an important contributing factor in degeneration of dopaminergic neurons in PD (Dias et al., 2013) and our data suggest that these injurious mechanisms may already be initiated early in life by perinatal inflammation. Whether these mechanisms are also important for synapse function later in life remains to be investigated.

#### Multiple Sclerosis

MS is characterized by pathological neuroinflammation, which results in a marked dysregulation of central nervous system functions. Cognitive dysfunction including lack of attention and reduced memory function is one of the common symptoms in MS patients (Mori et al., 2011). It is now recognized that the pathology in MS not only involves myelin defects but also injury to the gray matter (Geurts et al., 2012). Further, diffuse loss of synapses and synaptic dysfunction is observed in MS patients (Georgia et al., 2015). In hippocampal post-mortem tissue from MS patients, expression levels of several proteins that are important in synaptic maintenance (neurexin–neuroligin complex) and synaptic function (synaptophysin, synaptotagmin, postsynaptic density protein-95 and Ca^2+^-calmodulin-dependent protein kinase II) are decreased (Dutta et al., 2011). Notably, altered synaptic transmission can occur early in MS patients and in animal models of MS, (experimental autoimmune encephalomyelitis, EAE), prior to white matter injury (Ksiazek-Winiarek et al., 2015). Neurophysiological evidence points toward a link between neuroinflammatory episodes, synaptic hyperexcitability and neurodegeneration (Rossi et al., 2012). Inflammation also appears to be directly linked to synaptic dysfunction in MS via the complement system-mediated synapse elimination in the hippocampus (Michailidou et al., 2015). Pro-inflammatory cytokines, such as IL-1β, have also been correlated to the imbalance between GABAergic and glutamatergic transmission (Rossi et al., 2012) and consequently cognitive deficits in MS may occur as a result of cytokine-induced dysregulation of synaptic plasticity in the hippocampus (Nisticò et al., 2014; Di Filippo et al., 2015).

Taken together, synaptic disorganization and dysfunction is a common feature of neurodegenerative diseases. While there is evidence to suggest that neuroinflammatory challenges early in life predispose to PD, similar data are currently not available for AD and MS.

## Summary

**Figure 2:**
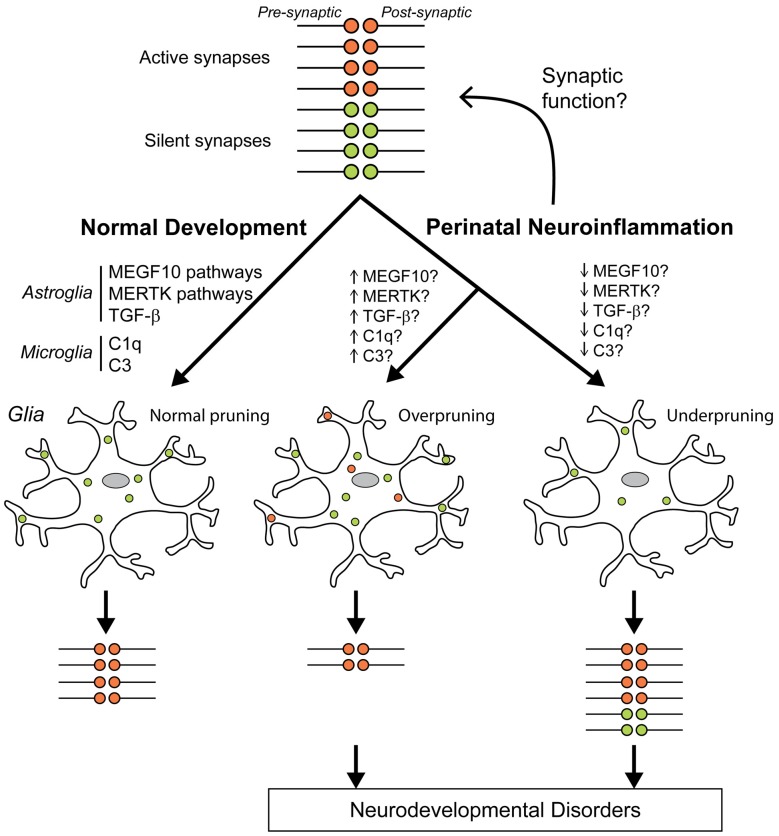
Hypothesis of how perinatal neuroinflammation can cause abnormal synapse pruning. We hypothesize that neuroinflammation at the time around birth dysregulates synaptic function and/or synaptic pruning that can lead to neurodevelopmental disorders. During normal development, silent (green) synapse terminals are appropriately pruned (phagocytosis) by glia cells (microglia and astrocytes) so that only active healthy (red) synapses remain (left). Complement protein 3 (C3), and C1q tag synapses for elimination by microglia during normal development and transforming growth factor (TGF)-β regulates C1q expression in neurons (Stevens et al., 2007; Bialas and Stevens, 2013). Astrocyte-mediated synapse pruning is complement-independent and is regulated by MEGF10 and MERTK pathways (Chung et al., 2013). We propose that perinatal neuroinflammation can directly stimulate/inhibit synaptic functions so that pruning rates by glial cells are adjusted and more/less synapses remain. Another possible pathological process is that neuroinflammation will change glia cells to a more pruning-prone phenotype resulting in overpruning so that some active healthy synapses are eliminated and fewer than normal synapses remain (middle) or to a reverse glial phenotype so that too many synapses remain (right).

Glia cells have emerged as important players in sculpting synapse structure and function, particularly during brain development. Neuroinflammation and disorganization of synapses are common features in several developmental and neurodegenerative disorders, suggesting that factors that affect glia cells during development may also have long-term effects on synapse function. The perinatal period is a vulnerable time in life where inflammatory challenges are of significance. However, the role of astrocytes and microglia in shaping the structure of the synapse and in the process of synapse pruning after perinatal challenges is still largely unknown. We propose that neuroinflammation during perinatal life changes the ability of glia cells to sculpt neuronal synapses, either by increasing or decreasing elimination of synapses (Figure 2). These changes may have important long-term effects on developmental psychiatric disorders as well as neurodegenerative disease.

## Author Contributions

All authors contributed to the text and figures.

## Conflict of Interest Statement

The authors declare that the research was conducted in the absence of any commercial or financial relationships that could be construed as a potential conflict of interest.

## Footnotes

^1^http://www.who.int/bulletin/volumes/88/1/08-062554/en
